# Association of microRNA 17 host gene variant (rs4284505) with susceptibility and severity of systemic lupus erythematosus

**DOI:** 10.1002/iid3.344

**Published:** 2020-08-27

**Authors:** Abdelhady R. Abdel‐Gawad, Sameerah Shaheen, Nouf A. Babteen, Eman A. Toraih, Rami M. Elshazli, Manal S. Fawzy, Nawal S. Gouda

**Affiliations:** ^1^ Department of Clinical and Chemical Pathology Sohag University Sohag Egypt; ^2^ Stem Cell Unit, Department of Anatomy, College of Medicine King Saud University Riyadh Saudi Arabia; ^3^ Department of Biochemistry, Faculty of Science University of Jeddah Jeddah Saudi Arabia; ^4^ Department of Surgery, School of Medicine Tulane University New Orleans Louisiana; ^5^ Genetics Unit, Department of Histology and Cell Biology, Faculty of Medicine Suez Canal University Ismailia Egypt; ^6^ Department of Biochemistry, Faculty of Physical Therapy Horus University ‐ Egypt New Damietta Egypt; ^7^ Department of Medical Biochemistry and Molecular Biology, Faculty of Medicine Suez Canal University Ismailia Egypt; ^8^ Department of Biochemistry, Faculty of Medicine Northern Border University Arar Saudi Arabia; ^9^ Department of Medical Microbiology and Immunology, Faculty of Medicine Mansoura University Mansoura Egypt; ^10^ Department of Microbiology, Faculty of Medicine Northern Border University Arar Saudi Arabia

**Keywords:** genetic polymorphisms, MIR17HG, rs4284505, systemic lupus erythematosus

## Abstract

**Objective:**

MicroRNAs are large family clusters of small noncoding RNAs that implicated in genetic and epigenetic regulation of several immunological processes and pathways. As an epigenetic modifier, the microRNA 17‐92 cluster host gene (MIR17HG) has been shown to regulate the expression of genes involved in systemic lupus erythematosus (SLE) pathway. This study aimed to explore the association of MIR17HG (rs4284505; A>G) variant with SLE development and phenotype in a sample of the Eastern Mediterranean population.

**Methods:**

A total of 326 participants (163 patients with SLE and 163 healthy controls) were enrolled in this study. The different genotypes of the MIR17HG (rs4284505) variant were characterized using the TaqMan real‐time polymerase chain reaction technique. Association with the available clinical and laboratory data, including the systemic lupus erythematosus disease activity index (SLEDAI), was also executed.

**Results:**

The MIR17HG (rs4284505*)* variant showed a protective effect against developing SLE under heterozygote (A/G vs A/A; odds ratio [OR] = 0.10, 95% confidence interval [CI] = 0.05‐0.20, *P* < 0.001) and dominant (A/G+G/G vs A/A; OR = 0.39, 95% CI = 0.25‐0.61, *P* < .001) models. This association was consistent even after SLE stratified by lupus nephritis. In contrast, rs4284505 (G/G) genotype conferred increased susceptibility to SLE (G/G vs A/A+A/G; OR = 2.15, 95% CI = 1.31‐3.53, *P* = .002). Moreover, the rs4284505 variant showed a statistically significant association with mucocutaneous lesions and SLEDAI scores (all *P* < .05).

**Conclusion:**

This study is the first one to explore that the MIR17HG rs4284505 is associated with SLE risk; (A/G) genotype conferred a protective effect, while the (G/G) genotype showed increased susceptibility to SLE and association with the disease severity in the study population.

## Graphical abstract

For the first time, this study showed that the microRNA 17‐92 cluster host gene (MIR17HG) is associated with the systemic lupus erythematosus (SLE) risk. The MIR17HG (A/G) genotype conferred a protective effect, while the (G/G) genotype showed increased susceptibility to SLE and association with the disease severity in a sample of the Eastern Mediterranean population.

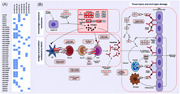

**Abbreviations**
ANA: antinuclear antibodies
Anti‐dsDNA: anti‐double‐stranded DNA antibodies
C3/4: complement 3/4
CI: confidence interval
MAF: minor allele frequency
MCV: mean cell volume
MIR17HG: microRNA 17‐92 cluster host gene
miRNAs: microRNAs
OR: odds ratio
PCA: principal component analysis
PCR: polymerase chain reaction
SLE: systemic lupus erythematosus
SLEDAI: systemic lupus erythematosus disease activity index
TF: transcription factor
WBCs: white blood cells

## 1 INTRODUCTION

The systematic lupus erythematosus (SLE) is a multisystem autoimmune disease with diverse clinical presentations.^1^ The pathogenesis of SLE is complex and undefined, with evidence of the interaction of several genetic and environmental causes.^2^

MicroRNAs (miRNAs) are short, evolutionary conserved, single‐stranded noncoding RNAs with approximately 22 nucleotides in length derived from large primary transcript encoded by their host genes.^3^ They have several genetic and epigenetic regulatory functions, modulating diverse cellular processes, and molecular pathways at the posttranscriptional level.^4^ The implication of miRNAs in immune response was evident in several studies,^5^, ^6^, ^7^, ^8^ and their deregulation was associated with multiple autoimmunity‐induced pathological entities, including SLE.^3^, ^9^, ^10^

Some “miRNAs‐containing” primary transcripts can yield a cluster of several and different miRNAs, rather than single miRNA, usually working in concert.^3^ One of the best‐studied miRNA clusters is the “miR‐17‐92 family,” which encoded by miR‐17 host gene (MIR17HG) and including miR‐17, miR‐18a, miR‐19a, miR‐20a, miR‐19b‐1, and miR‐92a‐1.^11^ This gene cluster is located on the plus strand of chromosome 13 (13q13.3) and spanned about 49 411 bases.^12^

**Figure 1:**
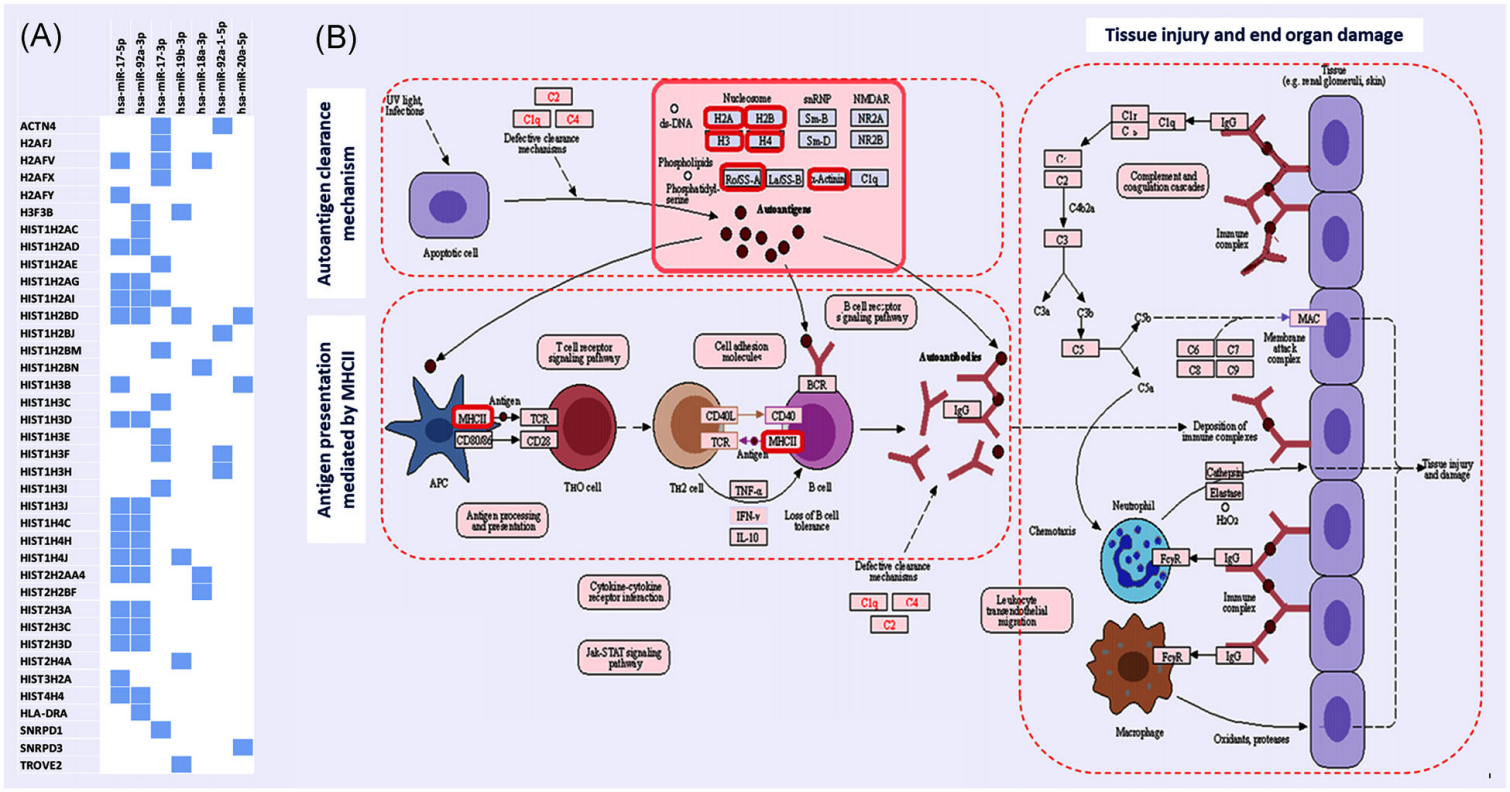
MIR17HG targets in systemic lupus erythematosus (SLE). SLE is an autoimmune disease featured by increased production of autoantibodies that are specific for self‐antigens, resulting in a wide range of clinical presentations. A, The miR‐17‐92 host gene family enclose a cluster of six microRNAs (miR‐17, miR‐18a, miR‐19a, miR‐20a, miR‐19b‐1, and miR‐92a‐1). Functional enrichment pathway analysis using Diana lab tools (http://diana.imis.athena-innovation.gr/DianaTools/index.php) revealed that these miRNAs could target at least 18 genes in the SLE pathway (KEGG ID: hsa05322, *P* = 1.179199e−43). B, The miR‐17‐92 cluster‐targeted genes (red‐colored boxes) code for the different histone family members (ie, H2A, H2B, H3, and H4), which are implicated in autoantigen clearance and tolerance mechanism and the major histocompatibility complex class II (MHCII), which is implicated in antigen presentation mediated by MHCII. All the previous stages of antigen processing and autoantibodies generation lead to tissue injury and end‐organ damage,^16^ (http://www.kegg.jp/kegg/kegg1.html). C3, complement 3; dsDNA, double‐stranded DNA; IFN‐γ, interferon‐γ; IgG, immunoglobulin G; IL‐10, interleukin 10; JAK‐STAT, Janus kinase‐signal transducer and activator of transcription; KEGG, Kyoto Encyclopedia of Genes and Genomes; MIR17HG, microRNA 17‐92 cluster host gene; NMDAR, *N*‐methyl‐d‐aspartate receptor; snRNP, small nuclear ribonucleoproteins; TCR, T cell receptor; TNF‐α, tumor necrosis factor‐α

Although this cluster was reported to be involved in cellular proliferation, angiogenesis, and survival,^4^, ^13^ other essential roles in multisystem processes, including the normal development, and the innate and acquired immune response among others, have been emerged.^11^, ^14^ Furthermore, the upregulation of this cluster in splenic T cells has been found to induce abnormal B cell activation and inflammatory T cell production by targeting the *Bim* gene in murine models of lupus.^15^ Our in silico analysis also revealed MIR17HG could target several immune‐related genes involved in SLE development and progression (Figure 1).^16^, ^17^, ^18^

As most genetic variants (ie, single‐nucleotide polymorphisms [SNPs]) related to SLE risk were identified to be located in noncoding regions of the genome,^19^ the study of MIR17HG cluster variants could enhance our understanding of its role in SLE pathogenesis. The most common SNP of this gene cluster is rs4284505 (G>A), which identified within the first intron of the MIR17HG. This variant has been associated with several diseases,^4^, ^12^, ^13^, ^20^, ^21^ but its impact on SLE development and severity has not been assessed. In this sense, the authors were inspired to investigate the association of MIR17HG (rs4284505) variant with the development of SLE and with the available clinical and laboratory data in a sample of the Middle Eastern population.

## 2 SUBJECTS AND METHODS

### 2.1 Study participants

This study is a case‐control study enrolled a total of 163 patients with SLE (147 [90.2%] females and 16 [9.8%] males), as well as 163 healthy controls (148 [90.8%] females and 15 [9.2%] males). The recruitment of the patients with SLE was settled from the Outpatient Clinics of the Rheumatology and Nephrology at the Suez Canal University Hospitals, Ismailia, Egypt. In addition, patients with SLE were categorized into 93 (57.1%) patients diagnosed with lupus nephritis and 70 (42.9%) patients diagnosed without lupus nephritis. The selection of patients with SLE was conducted according to the guidelines of the European League Against Rheumatism and the American College of Rheumatology.^22^ At the same time, the diagnosis of lupus nephritis was made as previously described.^23^ This study was conducted following the Helsinki Declaration guidelines, and the research ethics committee of the Faculty of Medicine, Suez Canal University, approved it. The controls were collected from the healthy blood donors attending the university hospital blood banks. They were matched in age and sex with patients with SLE. Individuals having a history of autoimmune illnesses, endocrine diseases, and any other chronic diseases were excluded. All enrolled subjects were asked to sign written consent before taking part. The basic characteristics and clinical features of patients with SLE were extracted from the medical health records. The assessment of disease activity was completed according to the systemic lupus erythematosus disease activity index (SLEDAI).^24^

### 2.2 Blood sampling and laboratory measurements

Upon establishing this study, 5 mm of peripheral blood were collected and allocated into two parts; the first part was gathered into a vacutainer tube containing ethylenediamine tetraacetic acid anticoagulant for purposes of genetic and hematological analysis, while the other part was centrifuged to isolate the serum for immunological and biochemical measurements. The estimation of complete blood count including hemoglobin, white blood cells (WBCs) count, neutrophil %, lymphocyte %, red blood cells count, hematocrit, mean cell volume (MCV), and platelet count was performed utilizing an automated cell counter (CELL‐DYN 1700; Abbott Diagnostics). The assessment of biochemical measurements involving complement 3 (C3), C4, C‐reactive protein, alanine transaminase, aspartate transaminase, creatinine, urea, and albumin was measured using automated biochemical analyzer Cobas c501 (Roche Diagnostics, Manheim, Germany). The indirect immunofluorescence technique was applied for the specification of antinuclear antibodies (ANA) and anti‐double‐stranded DNA antibodies (Anti‐dsDNA) (Bio‐Rad Laboratories, CA).

### 2.3 DNA extraction and allelic discrimination of MIR17HG rs4284505 variant

Genomic DNA from nucleated WBCs of whole blood was extracted utilizing the Mini Kit of QIAamp DNA extraction designated for blood samples (Catalog #51104; Qiagen, Hilden, Germany). The NanoDrop ND‐1000 spectrophotometer (NanoDrop Technologies, Inc, Wilmington, DE) was utilized for estimation of the quantity and purity of genomic DNA. The intronic variant (rs4284505; A>G) of MIR17HG was characterized and genotyped by the TaqMan allelic discrimination polymerase chain reaction (PCR) using StepOne Real‐Time (Applied Biosystems). The protocol used was managed blindly with a final volume of 20 μL containing 20 ng of DNA template, 1 μL of TaqMan SNP Genotyping Assay Mix (assays ID: C__26557482_10; Applied Biosystems, Waltham), and 10 μL of TaqMan Universal PCR Master Mix. Proper negative controls were applied in each run.^25^ About 10% of the samples were randomly selected for assay replication, and the results yield 100% concordance.

### 2.4 Statistical analysis

The processing of statistical analysis of the MIR17HG rs4284505 variant was performed using the SPSS program version 26.0 as well as R Language programming, version 4.0.0 and RStudio, version 1.3.959. The calculations of allelic and genotypic frequencies were executed using the Fischer exact test with *χ* ^2^, as previously described.^26^ The estimation of the Hardy‐Weinberg equation and the analysis of the MIR17HG rs4284505 variant among patients with SLE and healthy controls was computed utilizing SNPstats (www.SNPstats.org). Logistic regression analysis was assessed to determine the adjusted odds ratio (OR) with a 95% confidence interval (CI) of multiple genetic association models.^27^ The evaluation of principal component analysis (PCA) was conducted utilizing the “factoextra” and “FactoMineR” packages. *P* < .05 was adapted to be statistically significant.

## 3 RESULTS

### 3.1 Basic characteristics of the study population

The main characteristics of the SLE patients with and without lupus nephritis are summarized in Table 1. A total of 93 (57.1%) SLE patients with lupus nephritis (85 females and 8 males) and 70 (42.9%) SLE patients without lupus nephritis (62 females and 8 males) were included in this study. Their mean age (SD) at diagnosis was 36.4 (9.7) and 34.5 (9.4), respectively. Of the 163 patients with SLE, 58 (36.5%) had a positive family history of SLE. As depicted in Table 1, there was no significant difference regarding age at diagnosis (*P* = .21), sex (*P* = .60), positive family history (*P* = .74), and SLEDAI (*P* = .12). The clinical manifestations of SLE patients with and without lupus nephritis showed that only recurrent infection and ecchymosis were statistically significant (*P* < .05). The SLE patients with LN observed lower levels of albumin (mean = 4.26, SD = 0.60) vs SLE patients without LN (mean = 4.46, SD = 0.56), and *P* = .034. Furthermore, the analysis of immunological measurements showed that all patients with SLE were seropositive for ANA and anti‐dsDNA.

**Table 1:** Clinical characteristics of patients with systemic lupus erythematosus (SLE) stratified by presence of nephritis

	SLE without nephritis	Lupus nephritis	*P*
Total number	70	93	
Age at diagnosis	34.51 ± 9.39	36.43 ± 9.69	.21
Female	62 (88.6)	85 (91.4)	.60
Positive FH	26 (37.1)	32 (34.4)	.74
SLEDAI	2.84 ± 3.01	3.80 ± 3.64	.12
Clinical manifestations			
Mucocutaneous	70 (100)	90 (96.8)	.26
Musculoskeletal	31 (44.3)	54 (58.1)	.08
Recurrent Infection	10 (14.3)	34 (36.6)	**.002**
Pulmonary	39 (55.7)	49 (72.7)	.15
Neurology	39 (55.7)	42 (45.2)	.20
Weight loss	34 (48.6)	42 (45.2)	.75
Ecchymosis	3 (4.3)	16 (17.2)	**.013**
Laboratory results			
Hemoglobin, g/dL	11.62 ± 1.36	11.61 ± 3.62	.97
RBC (×10^6^/mm^3^)	4.14 ± 0.94	3.99 ± 0.68	.26
HCT (%)	38.37 ± 5.65	37.84 ± 6.34	.57
MCV, fL	81.51 ± 6.93	81.49 ± 5.90	.99
Platelet count (×10^3^/mm^3^)	255.54 ± 71.42	270.11 ± 81.35	.23
WBC (×10^3^/µL)	6.32 ± 2.25	6.72 ± 2.20	.26
Neutrophil (%)	61.97 ± 11.31	64.56 ± 9.52	.12
Lymphocyte (%)	31.79 ± 10.13	29.02 ± 9.77	.08
C3, mg/dL	92.24 ± 48.67	98.39 ± 48.25	.42
C4 mg/dL)	27.68 ± 15.87	28.25 ± 15.21	.82
CRP, mg/L	3.13 ± 3.64	2.74 ± 2.11	.42
ALT, U/L	26.73 ± 9.43	26.48 ± 9.73	.87
AST, U/L	27.00 ± 9.22	26.78 ± 8.48	.88
Serum creatinine, mg/dL	1.10 ± 0.70	1.21 ± 1.44	.52
Urea, mg/dL	34.45 ± 8.70	35.34 ± 13.81	.62
Serum albumin, g/dL	4.46 ± 0.56	4.26 ± 0.60	**.034**

*Note*: Data are presented as frequency (percentage) or mean ± SD. Bold values indicate significance at *P* < .05.

Abbreviations: ALT, alanine transaminase; AST, aspartate transaminase; C3, complement 3; CRP, C‐reactive protein; FH, family history of SLE; HCT, hematocrit; MCV, mean cell volume; RBC, red blood cell; SLEDAI, systemic lupus erythematosus disease activity index; WBC, white blood cell.

### 3.2 Genotypic and allelic frequencies of MIR17HG (rs4284505) variant

**Figure 2:**
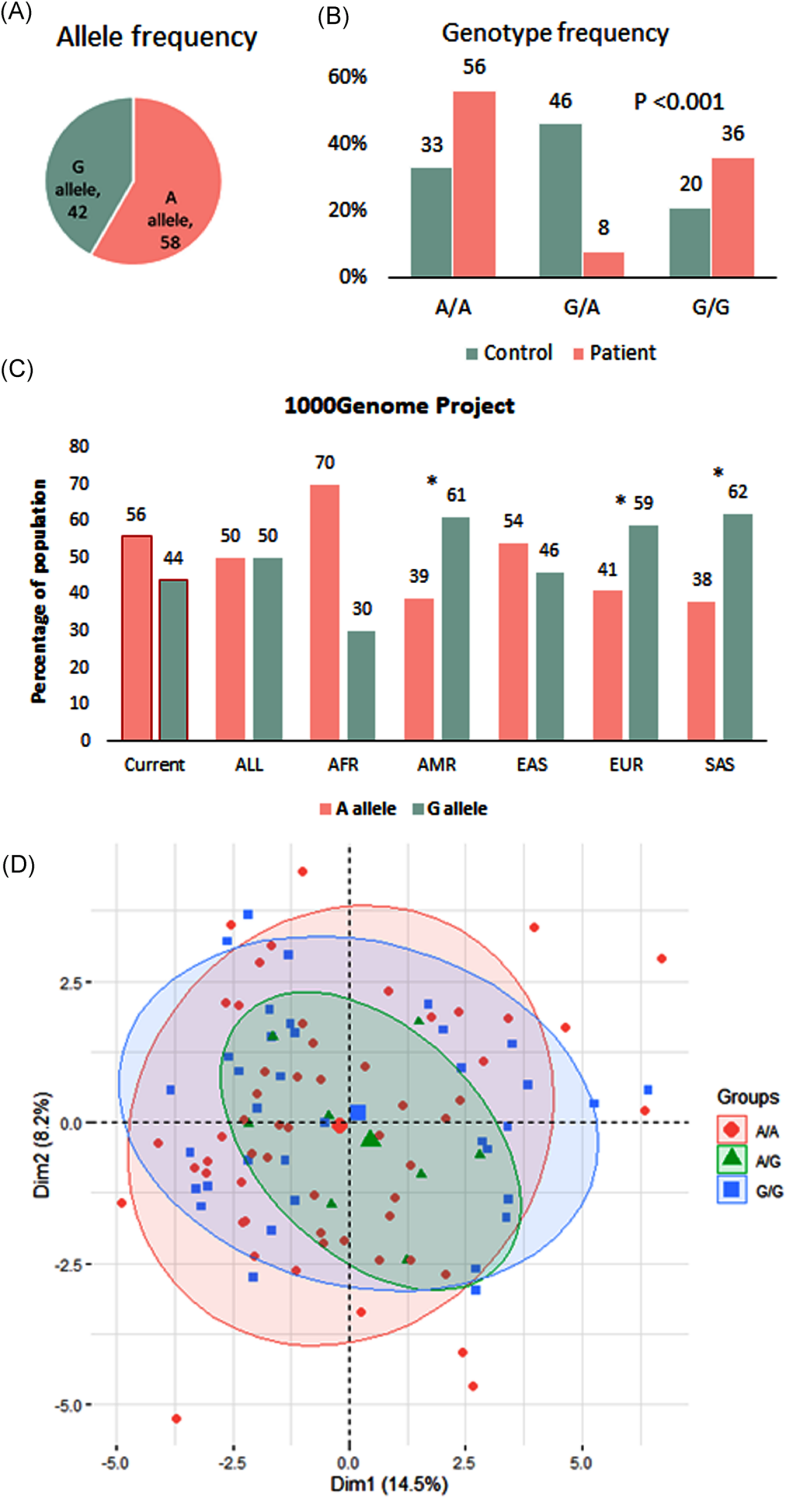
Genotype and allele frequencies in the study population. A, Pie chart for allele frequency in the whole study population. B, Genotype frequencies in SLE patients and controls. C, Allele frequencies of the MIR17HG (rs4284505) variant in the present and different populations. A *χ* ^2^ test was used. Statistical significance at *P < .05. Data source: 1000 genome project (https://www.internationalgenome.org/). D, Principle component analysis for data exploration. All variables in the study were used—the figure showing no clear distinct separation of SLE patients with different genotypes. AFR, Africa; AMR, America; EAS, East Asia; EUR, Europe; SAS, South Asia; SLE, systemic lupus erythematosus

The Hardy‐Weinberg equilibrium among healthy controls was in concurrence with observed equilibrium (*P* = .402) (Table 2). The minor allele frequency (MAF; G allele) of the MIR17HG variant was 0.40 among patients with SLE, while the MAF (G allele) among healthy controls was 0.44. Moreover, the common allele (A) frequency of the whole study population was 58%, while the MAF (G) was 42% (Figure 2A). The most predominant genotype among patients with SLE was A/A genotype, accounting for 56% of patients, while the most frequent genotype among healthy controls was G/A genotype, accounting for 46% of controls (Figure 2B). The MIR17HG allele frequencies in the present study were comparable with those related to the African and East Asian populations (Figure 2C). Upon construction, the PCA among patients with SLE, all variables showed no clear distinct demarcation of SLE patients with different genotypes (Figure 2D).

**Table 2:** Risk of systemic lupus erythematosus (SLE) by genetic association models of MIR17HG rs4284505 genotypes

Model	Genotypes	Healthy controls	Patients with SLE	Adjusted OR (95% CI)	*P*
Genotypic frequencies		n (%) 163	n (%) 163		
Codominant	A/A	54 (33.1)	91 (55.8)	1.0	**<.001**
	A/G	75 (46)	13 (8)	**0.10 (0.05‐0.20)**	
	G/G	34 (20.9)	59 (36.2)	1.03 (0.60‐1.76)	
Dominant	A/A	54 (33.1)	91 (55.8)	1.0	**<.001**
	A/G+G/G	109 (66.9)	72 (44.2)	**0.39 (0.25‐0.61)**	
Recessive	A/A+A/G	129 (79.1)	104 (63.8)	1.0	**.002**
	G/G	34 (20.9)	59 (36.2)	**2.15 (1.31‐3.53)**	
Overdominant	AA+GG	88 (54)	150 (92)	1.0	**<.001**
	A/G	75 (46)	13 (8)	**0.10 (0.05‐0.19)**	
Log‐additive	…	…	…	0.90 (0.70‐1.17)	.44
HWE		*χ* ^2^ = .*703, P* = .*402*			
Allelic frequencies		n (%) 326	n (%) 326		
Allelic	A	183 (56.1)	195 (59.8)	1.0	.38
	G	143 (43.9)	131 (40.2)	0.16 (0.85‐1.58)	

*Note*: Values are shown as the number (%). Adjusted covariates by age and sex. A *χ* ^2^ test was used. Bold values indicate that *P* < .05 and considered as statistically significant.

Abbreviations: 95% CI, 95% confidence interval; HWE, Hardy‐Weinberg equilibrium; OR, odds ratio.

### 3.3 Association of MIR17HG (rs4284505) variant with SLE development

The MIR17HG (rs4284505) variant conferred a protection against developing SLE under most genetic models, including heterozygote comparison (OR = 0.10, 95% CI = 0.05‐0.20, *P* < .001), dominant model (OR = 0.39, 95% CI = 0.25‐0.61, *P* < .001), and overdominant model (OR = 0.10, 95% CI = 0.05‐0.19, *P* < .001) (Table 2). Consistently, this association was kept even after stratifying the SLE cohort into patients with and without lupus nephritis (Table 3). Surprisingly, patients with SLE showed a significant difference of MIR17HG (rs4284505) variant with increased risk of developing SLE under recessive model (OR = 2.15, 95% CI = 1.31‐3.53, *P* = .002). On the contrary, upon adjusting covariates, age and sex, the allelic model of MIR17HG (rs4284505) variant indicated no significant association with disease risk (OR = 0.16, 95% CI = 0.85‐1.58, *P* = .38) (Table 2).

**Table 3:** Genetic association of MIR17HG rs4284505 variant with risk of systemic lupus erythematosus (SLE) stratified by the presence of lupus nephritis

		Healthy controls	Patients with SLE			
	Genotypes	n (%)	n (%)	*P*	Adjusted OR (95% CI)	*P*
Without nephritis	A/A		54 (33.1)	38 (54.2)	**<.001**	1.0
	A/G	75 (46)	6 (8.5)		**0.38 (0.19‐0.75)**	**.006**
	G/G	34 (20.9)	26 (37.1)		1.01 (0.50‐2.04)	.62
With nephritis	A/A	54 (33.1)	53 (56.9)	**<.001**	1.0	
	A/G	75 (46)	7 (7.5)		**0.34 (0.18‐0.64)**	**<.001**
	G/G	34 (20.9)	33 (35.4)		1.03 (0.60‐1.76)	.25

*Note*: Data are presented as number (percentage). A *χ* ^2^ test was used. Binary logistic regression analysis was performed to calculate the odds ratio (OR) and 95% confidence interval (CI) for heterozygote and homozygote comparison models adjusted by age and sex. Bold values indicate that *P*  < .05 and considered as statistically significant.

### 3.4 Association of MIR17HG (rs4284505) variant with clinical and laboratory parameters

The MIR17HG (rs4284505) variant was associated with mucocutaneous lesion (*P* = .021), SLEDAI (*P* = .023), and MCV (*P* = .046). There was no association with other clinical and laboratory markers (Tables 3 and 4).

**Table 4:** Association between MIR17HG rs4284505 genotypes and disease characteristics

		Total	A/A	G/A	G/G	*P*
Number			33	62	68	
Age at diagnosis	Mean ± SD	35.6 ± 9.6	34.97 ± 9.44	36.92 ± 9.76	36.85 ± 9.85	.38
Female	n (%)	147 (90.2)	80 (87.9)	13 (100)	54 (91.5)	.35
Organ involvement	Renal	93 (57.1)	50 (54.9)	6 (46.2)	37 (62.7)	.45
	Neurological	160 (98.2)	88 (96.7)	13 (100)	59 (100)	.29
	Mucocutaneous	85 (52.1)	39 (42.9)	7 (53.8)	39 (66.1)	**.021**
	Infection	44 (27)	25 (27.5)	3 (23.1)	16 (27.1)	.94
	Pulmonary	88 (54)	53 (58.2)	7 (53.8)	28 (47.5)	.43
	Musculoskeletal	81 (49.7)	41 (45.1)	10 (76.9)	30 (50.8)	.09
	Weight loss	76 (46.6)	48 (52.7)	5 (38.5)	23 (39)	.21
SLEDAI score	Mean ± SD	3.39 ± 3.41	2.68 ± 2.64	2.33 ± 3.17	3.98 ± 3.75	**.023**
Laboratory data	Hemoglobin, g/dL	11.6 ± 2.9	11.54 ± 1.79	11.42 ± 0.54	11.88 ± 4.23	.63
	RBC (×10^6^/mm^3^)	4.1 ± 0.7	4.09 ± 0.83	4.19 ± 0.62	4.09 ± 0.60	.99
	HCT (%)	38.0 ± 6.1	38.33 ± 6.49	37.08 ± 3.65	38.19 ± 5.82	.87
	MCV, fL	81.5 ± 6.3	81.94 ± 6.58	76.32 ± 5.43	81.67 ± 5.80	**.046**
	Platelet count (×10^3^/mm^3^)	263.9 ± 77.4	266.81 ± 87.10	271.42 ± 73.09	259.68 ± 62.97	.82
	WBC (×10^3^/µL)	6.5 ± 2.2	6.79 ± 2.12	6.29 ± 1.43	6.33 ± 2.48	.54
	Neutrophil (%)	63.4 ± 10.4	62.26 ± 10.75	65.75 ± 8.00	64.36 ± 10.40	.32
	Lymphocyte (%)	30.2 ± 10.0	31.23 ± 9.72	27.42 ± 8.78	28.73 ± 9.61	.08
	C3, mg/dL	96.3 ± 47.9	88.73 ± 47.49	114.46 ± 42.43	101.80 ± 48.28	.10
	C4, mg/dL	28.0 ± 15.5	27.78 ± 16.33	22.59 ± 9.97	29.25 ± 15.44	.45
	CRP, mg/L	2.4 (1.5‐3.2)	3.28 ± 3.66	2.38 ± 1.16	2.57 ± 1.45	.31
	ESR first hour, mm/hr	26.8 ± 13.6	26.80 ± 13.36	25.67 ± 11.72	27.15 ± 14.42	.66
	ESR second hour, mm/h	46.8 ± 21.8	50.11 ± 25.42	51.67 ± 23.75	49.83 ± 24.41	.97
	ALT, U/L	26.6 ± 9.6	27.07 ± 10.05	25.67 ± 10.22	26.12 ± 8.93	.64
	AST, U/L	26.9 ± 8.8	26.78 ± 8.41	24.92 ± 9.68	26.41 ± 8.45	.71
	Creatinine, mg/dL	1.0 (0.8‐1.2)	1.10 ± 1.00	1.10 ± 0.28	1.32 ± 1.52	.47
	Urea, mg/dL	35.0 ± 11.9	34.35 ± 9.11	33.33 ± 10.50	36.60 ± 15.31	.41
	Serum albumin, g/dL	4.3 ± 0.6	4.34 ± 0.56	4.18 ± 0.34	4.45 ± 0.58	.21

*Note*: Data are presented as frequency (percentage) or mean ± SD. *χ* ^2^ and one‐way ANOVA tests were used. Bold values indicate that *P* < .05 and considered as statistically significant.

Abbreviations: ANOVA, analysis of variance; ALT, alanine transaminase; AST, aspartate transaminase; C3, complement 3; CRP, C‐reactive protein; ESR, erythrocyte sedimentation rate; HCT, hematocrit; MCV, mean cell volume; RBC, red blood cell; SLEDAI, systemic lupus erythematosus disease activity index; WBC, white blood cell.

## 4 DISCUSSION

miRNAs are large family clusters of tiny noncoding RNAs that are implicated in genomic and epigenomic regulation.^20^ The primary function of miRNAs is to target the designated mRNAs causing their instability and/or degradation, mediating their inhibitory effects.^28^ Accumulating evidence identified various genetic variants within the MIR17HG gene, including (rs4284505; g.91349218A>G), (rs17735387; g.91353800G>T), (rs1428; g.91354516C>A), (rs7336610; g.91352883C>T), and (rs72640333; g.91352673T>A) that are destined to be correlated with different disorders involving multiple myeloma,^13^ breast cancer,^4^ multiple sclerosis,^21^ and periapical lesions.^20^

Until now, to the best of our knowledge, this is the first study investigating the association of MIR17HG (rs4284505; A>G) variant with the development of SLE among a sample of Middle Eastern population. Our findings indicated that the A/G genotype revealed a protective effect against the development of SLE, and this protective effect remains significant after stratifying the patients according to the presence of lupus nephritis. In contrast, the G/G genotype conferred an increased susceptibility of SLE development. Interestingly, the substitution of A to G has been found to disrupt a regulatory region upstream to the MIR17HG cluster, affecting multiple transcription factors (TF)‐binding elements (Table S1) with subsequent change in the type of activated genes. In this sense, having activated TF for genes with A as well as genes with G allele simultaneously might synergistically promote the expression of protective genes, and A/G genotype carriers become less susceptible to the disease. In contrast, having two copies of the G allele might promote the expression of genes that increase susceptibility to the disease and increase the severity. Moreover, due to the functional relationship of the intronic miRNA (eg, MIR17HG) with its host gene, this miRNA family may have an individual negative regulatory feedback by directly targeting and regulating the host transcripts, mediating some roles in disease phenotype at the transcriptomic level.^29^ Further functional studies will be required to confirm these findings.

Accumulating evidence has uncovered the potential role of the MIR17HG cluster with the macrophage colony‐stimulating factor in promoting CD34^+^ hematopoietic progenitor cell differentiation into monocytes in human cord blood.^30^ In addition, the upregulation of this cluster in the DN1 stage has been associated with increased proliferation and survival of CD4^+^ T cells, which mediates the autoimmunity.^14^ By targeting *BIM* and *PTEN* genes, which play a critical role in immune tolerance mechanism, in B/T lymphocyte precursors and memory CD8^+^ T cell, respectively, miR‐17‐92 family was associated with B and T cell development and autoimmunity.^31^, ^32^

MIR17HG transcriptomic signature also was reported to be involved in the pro‐B cell transition to pre‐B regulating B cells generated in the bone marrow during the early stages of development.^3^ By analogy to other types of autoimmune disease, earlier studies also support the key role MIR17HG cluster play in controlling the inflammatory process and disease activity through regulation of the “Apoptosis Signal‐Regulating Kinase 1 (ASK1) signalosome,” a central actor in the inflammatory pathways activated during rheumatoid arthritis.^33^, ^34^ Collectively, all the above evidence confirm the role which MIR17HG play in autoimmunity and tissue damage (Figure 1), supporting the potential association of the specified genetic variant with SLE development and severity, as reflected in association of the risky genotype (GG) with a higher frequency of mucocutaneous manifestations and a higher SLEDAI score than the other genotypes in the study population.

The current work also observed that the MAF of rs4284505*G accounted for 42% of the study population. Comparing with the 1000 genome project indicated that our MAF has resembled with East Asian population (46%), but entirely disagreed with African (30%), American (61%), European (59%), and South Asian (62%).

Similarly, a recent report studied the association of MIR17HG (rs4284505) variant with the susceptibility for multiple myeloma, revealed that the G/G genotype observed increased risk with the developing of multiple myeloma and the A/G genotype revealed a poor prognosis with the outcome of the disease.^13^ On the contrary, another study investigated the association of MIR17HG (rs4284505) variant with the risk of persistent apical periodontitis and observed no significant association of this variant under recessive and dominant models with the progression of the disease.^20^ Also, we encountered a single study that revealed a protective effect of the MIR17HG rs4284505*A allele with the development of breast carcinoma among the Australian population.^4^ These conflicting findings confirm the previous notion that the genetic variants often exert their effect in “a cell‐type‐specific and context‐dependent manner.”^19^, ^35^, ^36^ Additional contributing factors could be due to the ethnicity, environmental, and geographical factors, as well as the variability in the sample size and methodology of the different studies.

## 5 CONCLUSION

In conclusion, the MIR17HG (rs4284505) variant was associated with SLE development among Egyptian subjects. The MIR17HG A/G genotype conferred a protective effect with the progression of SLE, while the MIR17HG G/G genotype associated with increased susceptibility to SLE development and severity.

## CONFLICT OF INTERESTS

The authors declare that there are no conflict of interests.

## AUTHOR CONTRIBUTIONS

All listed authors have made substantial contributions to conception/design, acquisition of data, analysis of data, and have been involved in drafting the manuscript or critically revising for content. All authors have given final approval of the version to be published, taken responsibility for the content, and agreed to be accountable and ensure accuracy of the work.

## ETHICS STATEMENT

All procedures performed in studies involving human participants were following the ethical standards of the institutional and/or national research committee and with the 1964 Helsinki declaration and its later amendments or comparable ethical standards.

## Supporting information

Supporting information

## Data Availability

All data generated or analyzed during this study are included in this submitted article.
