# Supplementary material for: Association of microRNA 17 host gene variant (rs4284505) with susceptibility and severity of systemic lupus erythematosus
Source: Immun Inflamm Dis. 2020 Aug 27;8(4):595–604. doi: 10.1002/iid3.344 (PMC7654399; doi:10.1002/iid3.344)
Supplement: Supplementary file 1 — Supporting information [file IID3-8-595-s001.pdf]

	A	B	C	D
1	**Supplementary Table S1. Functional impact of MIR17HG polymorphism on the regulatory regions**			
2	**Consequence type**	**Transcription factors**	**Motif positio**	**Motif score change**
3	TF binding site	FOXO1::ELF1, FOXJ2::ELF1, FOXJ3::ELF1	13 (out of 15)	Less like consensus sequence
4	TF binding site	ELF1, ELF4, ELF5, ELF3, ELF2, ETV7	3 (out of 12)	More like consensus sequence
5	TF binding site	ETV2::DLX3, HOXB2::ELF1, HOXB2::ELK3	9 (out of 18)	Less like consensus sequence
6	TF binding site	E2F1::ELK1	14 (out of 22)	Less like consensus sequence
7	TF binding site	ETV2::PAX5, ELK1::PAX1, ELK1::PAX5, ELK1::PAX9	1 (out of 20)	Less like consensus sequence
8	TF binding site	HOXB2::ELF1	8 (out of 17)	Less like consensus sequence
9	TF binding site	ETV2::HOXA2, FLI1::DLX2, HOXB2::ELF1, HOXB2::ELK3, ETV2::DRGX, ELK1::HOXA1, FLI1::DRGX, ETV5::DRGX, ETV5::HOXA2, HOXB2::ELK1	1 (out of 14)	Less like consensus sequence
10	TF binding site	HOXB2::ELF1	13 (out of 22)	More like consensus sequence
11	TF binding site	HOXA3::PAX5	14 (out of 20)	Less like consensus sequence
12				
13	Motif position is the position of the variant within the motif, in base pair coordinates, from the start of the motif.			
14	The motif scores are between 0 and 1. It indicates how strong a binding site it is for the transcription factor, with 1 the best and 0 the worst.			
15	The score change indicates whether the score increases or decreases and by how much as a result of the SNP. It indicates whether the SNP changes			
16	**Data source:** ensembl.org			
